# Safety and efficacy of glibenclamide combined with rtPA in acute cerebral ischemia with occlusion/stenosis of anterior circulation (SE-GRACE): study protocol for a randomized controlled trial

**DOI:** 10.1186/s12883-020-01823-z

**Published:** 2020-06-11

**Authors:** Kaibin Huang, Zhong Ji, Yongming Wu, Yunqiang Huang, Guangning Li, Saijun Zhou, Zhi Yang, Wenguo Huang, Guoshuai Yang, Guohu Weng, Pingyan Chen, Suyue Pan

**Affiliations:** 1 Department of Neurology, Nanfang Hospital, Southern Medical University, Guangzhou North Avenue 1838#, Guangzhou, 510515 China; 2 Department of Neurology, Heyuan People’s Hospital, Heyuan, China; 3 Department of Neurology, Huadu district People’s Hospital, Guangzhou, China; 4 Department of Neurology, The First Affiliated Hospital of Wenzhou Medical University, Wenzhou, China; 5 Department of Neurology, Maoming People’s Hospital, Maoming, China; 6 Department of Neurology, Maoming Hospital of Traditional Chinese Medicine, Maoming, China; 7 Department of Neurology, Haikou People’s Hospital, Haikou, China; 8 Department of Neurology, Hainan Hospital of Traditional Chinese Medicine, Haikou, China; 9 Department of Biostatistics, School of Public Health, Southern Medical University, Guangzhou, China

**Keywords:** Combined treatment, Glibenclamide, Randomized controlled trial, Stroke

## Abstract

**Background:**

Thrombolysis with recombinant tissue plasminogen activator (rtPA) improves outcome for patients with acute ischemic stroke (AIS), but many of them still have substantial disability. Glibenclamide (US adopted name, glyburide), a long-acting sulfonylurea, shows promising result in treating AIS from both preclinical and clinical studies. This study investigates the safety and efficacy of glibenclamide combined with rtPA in treating AIS patients.

**Methods:**

This is a prospective, randomized, double-blind, placebo-controlled, multicenter trial with an estimated sample size of 306 cases, starting in January 2018. Patients aged 18 to 74 years, presented with a symptomatic anterior circulation occlusion with a deficit on the NIHSS of 4 to 25 points and treated with intravenous rtPA within the first 4.5 h of their clinical onsets, are eligible for participation in this study. The target time from the onset of symptoms to receive the study drug is of 10 h. Subjects are randomized 1: 1 to receive glibenclamide or placebo with a loading dose of 1.25 mg, followed by 0.625 mg every 8 h for total 5 days. The primary efficacy endpoint is 90-day good outcome, measured as modified Rankin Scale of 0 to 2. Safety outcomes are all-cause 30-day mortality and early neurological deterioration, with a focus on cardiac- and glucose-related serious adverse events.

**Discussion:**

This study will provide valuable information about the safety and efficacy of oral glibenclamide for AIS patients treated with rtPA. This would bring benefits to a large number of patients if the agent is proved to be effective.

**Trial registration:**

The trial was registered on September 14th 2017 at www.clinicaltrials.gov having identifier NCT03284463. Registration was performed before recruitment was initiated.

## Background

Stroke is the second leading cause of death worldwide [1], and the global burden attributable to stroke is still increasing [2]. Thrombolytic therapy with intravenous recombinant tissue plasminogen activator (rtPA), aiming at early reperfusion, has been proven effective for patients with acute ischemic stroke (AIS) when they are treated within 4.5 h of stroke onset [3]. However, the number of patients eligible for the treatment with rtPA is limited because of the restricted time window [3]. In addition, the likelihood of complete early recanalization in proximal occlusion is still unsatisfying [4], and thrombolytic therapy increases the risk of symptomatic intracranial hemorrhage by 3.75 folds and early death by 1.69 folds [3]. Combined treatment upon intravenous rtPA thrombolysis is necessary to reduce its side effects and meanwhile to bring more overall benefits.

Recent advance in endovascular therapy has greatly increased the likelihood of recanalization and thereby improved functional outcome. Compared with medical care alone, endovascular thrombectomy as add-on to intravenous thrombolysis performed within 6 to 8 h after large vessel ischemic stroke in the anterior circulation provides beneficial functional outcomes, without increased detrimental effects [5]. Current clinical guidelines has recommended endovascular therapy for eligible patients with acute ischemic stroke [6]. Nevertheless, about 56% of patients remain to experience poor outcome even after endovascular treatment [5]. Hence, the search for additional treatment on the basis of rtPA thrombolysis and endovascular thrombectomy should be ongoing.

Glibenclamide (US adopted name, glyburide), a long-acting sulfonylurea, has been used safely for management of type 2 diabetes for decades. Recent researches showed that glibenclamide significantly reduced mortality, brain swelling, as well as improved neurological function in different animal models of stroke [7, 8]. More significantly, glibenclamide was also found to be effective in a clinically relevant rodent model of stroke treated with rtPA, and glibenclamide was said to have a therapeutic window of 10 h after the onset of ischemia [9]. In human, type 2 diabetes patients taking sulfonylurea agents such as glibenclamide both at the time of stroke and during hospitalization tended to have better functional outcome and less hemorrhagic transformation [10, 11]. A well designed prospective study (GAMES-RP) in patients with large anterior circulation infarction also presented a protective role of glibenclamide in alleviating brain swelling and blood-brain barrier disruption [12]. Together, these studies provide strong evidence that glibenclamide may be suitable to combine with rtPA thrombolysis [13].

Based on these data, we aim to conduct this trial to investigate the safety and efficacy of glibenclamide combined with rtPA in the treatment of patients with acute anterior circulation infarction.

## Methods

### Design

The Safety and Efficacy of Glibenclamide combined with rtPA in Acute Cerebral Ischemia with occlusion/stenosis of anterior circulation (SE-GRACE) study is a multicenter, randomized, double-blind, placebo-controlled, 1:1 parallel-group trial. The active comparison is glibenclamide versus a placebo. The treatment is provided in addition to intravenous rtPA thrombolysis within the first 4.5 h of the onset of symptoms plus best medical management according to national and international guidelines [14], and may include endovascular treatment. The study will be run in 8 large academic hospitals having experience in conducting such clinical trials in China (Additional file 1). Enrollment will begin in January 2018 and expect to end in September 2020. This trial has been approved by the Medical Ethics Committee of Nanfang Hospital (NFEC-2017-130) and all participating institutions have obtained Local Ethics Committee approval before trial initiation. The SE-GRACE study is registered at the clinicaltrials.gov (identifier NCT03284462).

### Patient inclusion and exclusion criteria

Eligibility criteria are presented in Table 1. Patients aged 18 to 74 years with AIS and a symptomatic anterior circulation occlusion with a deficit on the National Institute of Health Stroke Scale (NIHSS) of 4 to 25 points, treated with intravenous rtPA within the first 4.5 h of clinical onset, are eligible for participation in this trial. The exclusion criteria are modified from that set in the previous study [15]. The diagnosis of AIS is based on cranial CT scan and clinical manifestations. However, since not all patients will receive brain MRI examination or angiography before randomization, the diagnosis of anterior circulation AIS will base largely on clinical symptoms and signs, and thus some patients with posterior circulation occlusion alone may be mistakenly included before an MRI examination or angiography done. Therefore, once these subjects are diagnosed with posterior circulation occlusion alone after being included, they would stop using the study drugs immediately, but continue to receive other standard treatment and follow-up. These participants will be excluded from the modified intent-to-treat population when performing statistical analysis. Site investigators are responsible for enrolling participants, obtaining informed consent and managing randomization.

**Table 1:** Inclusion and exclusion criteria for the SE-GRACE trial

Criteria	Description
Inclusion criteria	1. Clinical diagnosis of acute ischemic stroke in the MCA territory (PCA and/or ACA territory involvement in addition to primary MCA territory stroke is acceptable)
	2. Aged ≥18 and ≤ 74 years
	3. A baseline NIHSS score between 4 to 25
	4. Intravenous rt-PA thrombolysis conducted within 4.5 h after stroke onset, if known, or the time last seen well [termed “time last known at neurologic baseline” (TLK@B)]
	5. The time to the start of administration of Study Drug must be ≤10 h after time of symptom onset or TLK@B
	6. Informed consent was signed by the subject or the legal representative
Exclusion criteria	1. Prior to stroke, significant disability exist, with modified Rankin Scale > 1 point
	2. With medical history or evidence of cerebral hemorrhage, subarachnoid hemorrhage, arteriovenous malformation, cerebral aneurysm or brain tumor
	3. With clinical or imaging evidence of contralateral cerebral infarction which is believed to have influence on the patient outcome by the investigators
	4. With clinical or imaging evidence of occlusion in vertebral or basilar artery
	5. With clinical evidence of brain herniation, e.g., one or two dilated, fixed pupils; unconsciousness (i.e., C2 on item 1a on the NIHSS); and/or loss of other brainstem reflexes, attributable to edema or herniation according to the investigator’s judgment
	6. With gastrointestinal bleeding and instable hemodynamics or other causes that force the patient to stop nutritional support
	7. Renal disorder from the patient’s history (e.g., dialysis) or eGFR of < 60 mL/min/1.73 m^2^
	8. Severe liver disease, or ALT > 3 times upper limit of normal or bilirubin > 2 times normal (subjects may be randomized if liver function tests have been drawn but are not yet available and the subject has no known history of liver disease; however treatment with Study Drug cannot commence until liver function tests are available and indicate ALT > 3 times upper limit of normal and bilirubin B2 times upper limit of normal)
	9. Blood glucose < 3.0 mmol/L at enrollment or immediately prior to administration of Study Drug, or a clinically significant history of hypoglycemia
	10. Acute ST elevation myocardial infarction, and/or acute decompensated heart failure, and/or Tc > 520 ms, and/or known history of cardiac arrest (PEA, VT, VF, asystole), and/or admission for an acute coronary syndrome, myocardial infarction, or coronary intervention within the past 3 months
	11. Known sulfonylurea treatment within 7 days. Sulfonylureas include glibenclamide/glyburide; glibenclamide plus metformin; Xiaoke Pill (a Chinese patent medicine with main effective constituent of glibenclamide); glimepiride; repaglinide; nateglinide; glipizide; gliclazide; tolbutamide; glibornuride
	12. Known treatment with bosentan within 7 days
	13. Known allergy to sulfa or specific allergy to sulfonylurea drugs
	14. Known G6PD enzyme deficiency
	15. Pregnant women. Women must be either postmenopausal (as confirmed by the LAR), permanently sterilized or, if ≤50 years old must have a negative test for pregnancy obtained before enrollment
	16. Breast-feeding women who do not agree (or their LAR does not agree) to stop breastfeeding during Study Drug infusion and for 7 days following the end of Study Drug infusion
	17. Patients already enrolled in a non-observation-only stroke study, or with life-expectancy < 6 months not related to current stroke, or those unlikely to be compliant with follow up
	18. Patients currently receiving an investigational drug
	19. Mentally incompetent (prior to qualifying stroke) patients and wards of the state
	20. Patients who, in the opinion of the investigator, are not suitable for the study (reason to be documented)

### Randomization and blinding

Eligible patients will be allocated using a web-based 1:1 randomization process. The randomization will be stratified by center, and the Pocock and Simon’s minimization method will be implemented to balance two important prognostic factors, endovascular treatment and baseline NIHSS score (≥14 or < 14). The study drugs, glibenclamide and placebo, are manufactured by Succhi Pharmaceutical (Zhongshan, China) following Good Manufacturing Practices regulations, and are coded and concealed by an independent biostatistician. In this manner, all trial participants, care providers and outcome assessors will be blinded to intervention allocation.

### Treatment

**Fig. 1:**
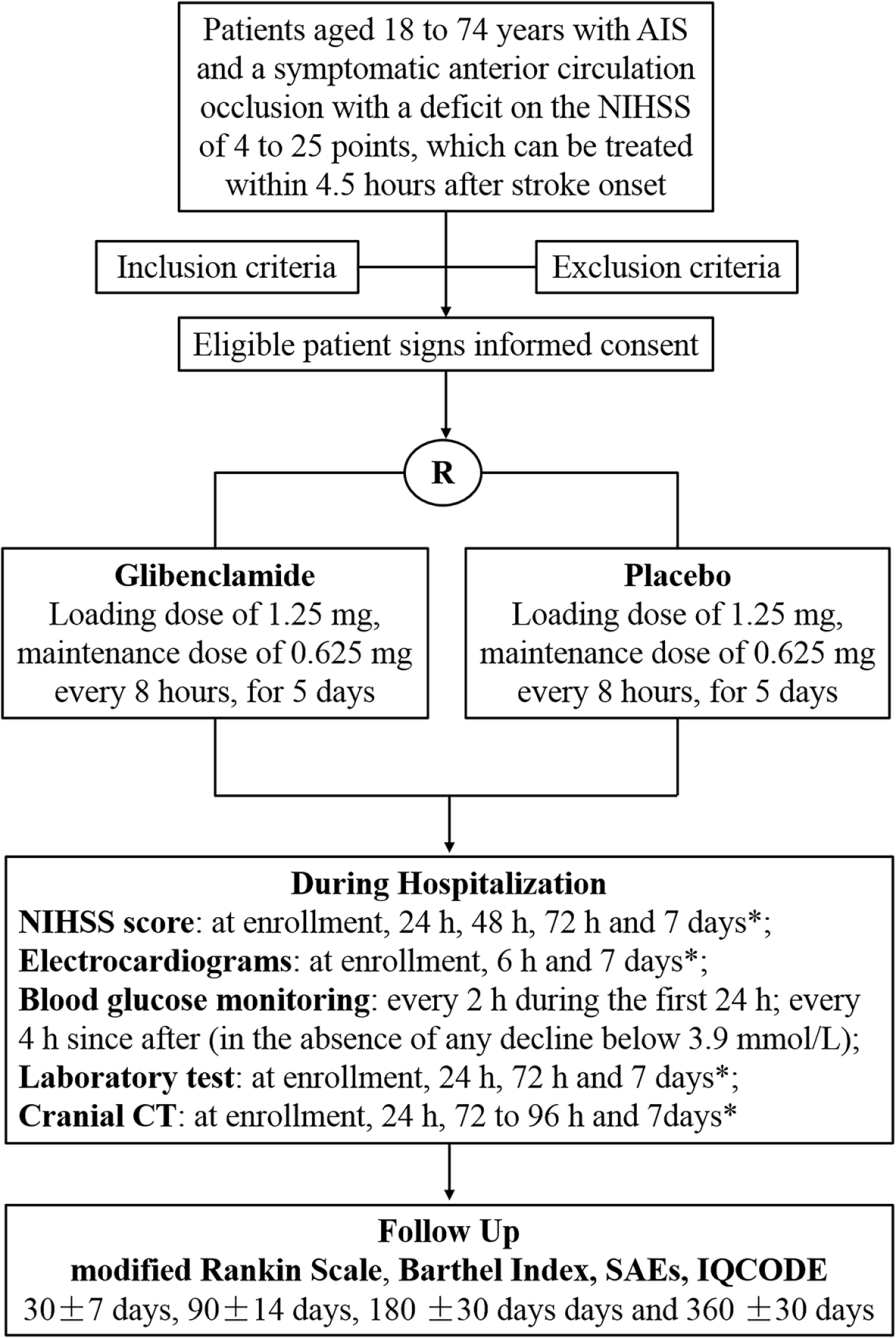
Flowchart of the trial. AIS, acute ischemic stroke; SAEs, severe adverse events; IQCODE, Informant Questionnaire on Cognitive Decline in the Elderly. *If a patient is discharged within 7 days, the examination will be performed at the day of discharge

An overview of the study flowchart is presented in Fig. 1. Intravenous rtPA treatment will be initiated at a standard dose and regimen (0.9 mg/kg, initial bolus of 10% of the total dose and the remaining dose as an intravenous infusion lasting over 60 min) following current clinical guidelines [14]. When a patient meets all the inclusion/exclusion criteria and signs the informed consent, randomization will be performed and the study drug will be given as soon as possible after starting the infusion of rtPA treatment. Based on previous studies, the target time from symptom onset to the start of study drug is ≤10 h [9, 15]. The study drug (glibenclamide/placebo, 2.5 mg/pill) will be evenly divided with a pill splitter and given orally by trained hospital nurses. If the patient is unable to ingest orally due to dysphagia or altered consciousness, the study drug will be grinded and dissolved with water and administered through a nasogastric tube, followed by flushing down the nasogastric tube with water. In order to achieve a steady-state concentration rapidly, a loading dose of 1.25 mg will be given, followed by 0.625 mg every 8 h for 5 days. The total given dose will be 10 mg. Since the optimal dosage of oral glibenclamide in treating AIS remains unclear, in this study we use a relatively low-dose regimen differed from that of intravenous preparations [16], with combined consideration of its high bioavailability and hypoglycemic potency. In our preliminary study, the current dose regimen of oral glibenclamide was well tolerated, associated with less brain edema, and did not increase the risks of early death, hypoglycemia, and early neurological deterioration [17]. In addition, our unpublished data has shown that this dosing regimen was able to result in average steady-state plasma glibenclamide levels of 19 ng/mL, which is not inferior to the concentrations reached by the effective therapeutic dose in rats [16].

To increase the adherence to intervention protocols, all participants will receive their study drugs at the scheduled time and take the medicine under the supervision of trained nurses. Because all subjects will receive thrombolytic therapy, for safety reasons, these patients will be hospitalized for minimum of 5 days to monitor the changes in their conditions with their complete examinations such as stroke etiology and vascular status. These all will be undertaken unless the patients voluntarily request discharge or death occurs. The patients who ask to be released from the hospital within 5 days will no longer be treated with the study drugs but will be included in the modified intent-to-treat population.

Neurological statue presented as NIHSS scores will be assessed at enrollment and at 24, 48, 72 h and 7 days. Electrocardiograms will be obtained at baseline, 6 h after the study drug administration, and at 7 days. Vital signs will be monitored frequently during the period of study drug administration. Safety lab values will be assessed at enrollment and at 24, 48, 72 h and 7 days. Cranial CT examination will be performed before randomization and repeated at 24 h, 72 to 96 h, and as clinical indicated. If a participant is discharged within 7 days, the examination will be performed at the day of discharge instead.

Since glibenclamide is an active hypoglycemic agent, blood glucose levels will be checked every 2 h during the first 24 h, followed by every 4 h in the absence of any decline below 3.9 mmol/L during the period of study drug administration. In the case of hypoglycemia, blood glucose level will be corrected by administration of glucose or glucagon, as appropriate. For safety reason, if two consecutive events of hypoglycemia (< 3.9 mmol/L) or one incidence of serious hypoglycemia (< 2.2 mmol/L) occurs, the study drug will be suspended. Blood glucose and corrective glucose administration will be reported.

Study participants will receive standard care and therapy for stroke, as well as other appropriate therapies according to clinical guidelines from the Chinese Medical Association and the American Heart Association/American Stroke Association [14, 18]. Endovascular therapy could be considered when the participant meets the criteria for endovascular therapy and operates according to the current clinical guideline [14]. Nutrition support in the form of early enteral nutrition will be initiated within 24–48 h in general, and, when indicated, parenteral nutrition will be considered, following the corresponding guidelines [14, 19]. Bolus osmotherapy is reserved for patients with clinical deterioration or radiological evidence of significant midline shift and/or mass effect. In the condition of malignant middle cerebral artery occlusion with evidence of clinical deterioration due to mass effect, indication of decompressive craniectomy will be judged by neurosurgeons but the final decision will be left to the legal representatives [20].

During the whole study period, if a suspected unexpected serious adverse reaction (SUSAR) is found, the sponsor and the principal investigator of this study have the authority and ability to unseal the patient’s intervention allocation, through the biostatistician responsible for the trial website.

### Outcome assessments

All patients will be followed up by trained neurologists blinded to study data. The primary and secondary endpoints of this study are summarized in Table 2. The SE-GRACE trial is designed with combination of clinical, imaging and biochemical outcomes to provide information about clinical benefits and relevant mechanism of action. Subjects will be followed up in person or through telephone review at 30 ± 7 days, 90 ± 14 days (the primary end-point), 180 ± 30 days and 360 ± 30 days after the onset of AIS, using modified Rankin Scale (mRS), Barthel Index (BI) and Informant Questionnaire on Cognitive Decline in the Elderly (IQCODE) (Fig. 1) [21, 22]. The primary outcome will compare the proportion of “good outcome” in the glibenclamide-treated group to the placebo group, with good outcome defined as a patient with a mRS of 0–2 at 90 days after the onset. The BI is a 10-item measure of activities of daily living which is frequently used in clinical practice and as a trial outcome measure in stroke, with a total score of 60 to 100 generally regarded as good outcome [23]. The IQCODE is an informant-based assessment tool for diagnosis of poststroke dementia and/or multidomain cognitive impairment, with a pooled sensitivity of 0.81 and specificity of 0.83 in a recent a systematic review [22]. The cutoff value of IQCODE for diagnosing poststroke dementia is generally considered to be > 3.4 [21, 22].

**Table 2:** Primary and secondary endpoints of the trial

Outcome	Description
Primary outcome	The ratio of mRS ≤ 2 at 90 days after the stroke onset
Secondary outcome	1. The ratio of NIHSS decreased ≥4 points at 7 days after the stroke onset
	2. The ratio of parenchymal hemorrhagic transformation in cranial CT within 96 h after the onset
	3. The ratio of midline shift ≥6 mm in cranial CT within 96 h after the onset
	4. The mRS shift at 90 days after the onset
	5. The ratio of Barthel Index of 60–100 points at 90 days after the onset
	6. The proportion of Informant Questionnaire on Cognitive Decline in the Elderly (IQCODE) of ≤3.40 at 6 months and 1 year after the stroke onset
	7. The serum concentration of MMP-9 at baseline, and at 24, 48, and 72 h after onset
Safety outcome	1. The mortality at 90 days after the onset
	2. The ratio of neurological deterioration (NIHSS increased ≥4 points) within 24 h after the onset
	3. The incidence of hypoglycemia (random blood glucose < 3.9 mmol/L)
	4. The incidence of cardiac events in cardiac examination (ECG, echocardiography)
	5. The incidence of pulmonary infection
	6. The incidence of adverse event and serious adverse event

In terms of cranial CT, parenchymal hemorrhagic transformation (PH) including PH1 type and PH2 type are defined as hematoma with some space-occupying effect [24], which has been considered as a bad prognostic sign [25]. Midline shift will be measured in the section of third ventricle and categorized at 6 mm. The serum concentration of matrix metalloproteinase-9 (MMP-9) will be measured at baseline, and at 24, 48, and 72 h after the stroke onset to reflect the integrity of the blood-brain barrier [15].

Since the study will recruit AIS with NIHSS scores of 4 to 25 who are treated with intravenous rtPA, most of the included patients are severely ill and thereby most adverse events (AE) are common in this patient group irrespective of treatment strategies. AEs will be recorded on daily basis. AEs relevant to the study drug are considered to be the need for decompressive craniectomy (determined by neurosurgeons) and hypoglycemia < 3.9 mmol/L in the first 5 days. All-cause mortality, cardiac mortality, early neurological deterioration (NIHSS increased ≥4 points within 24 h of the onset) and cardiac-related and blood glucose-related AEs/SAEs will be of specific focus.

### Quality control and data monitoring

The Executive Committee (12 members, lead investigator - Suyue Pan) is responsible for the development of the study protocol and its amendments. The protocol is adapted to the ‘Standard Protocol Items: Recommendations for Interventional Trials’ (SPIRIT), and an additional file is added listing each paragraph and its location in the protocol (Additional file 2).

All investigators are “good clinical practice” (GCP) certified personnel and have participated in the training sessions to ensure that they fully understand the trial protocols and the standard procedures before the study. Other involved personnel (i.e. nurses) have been trained in their specific roles. Inspectors will be nominated to visit each study site regularly in order to monitor the implementation of the protocols and assess the compliance of the participants and investigators.

All data will be recorded in case report form (CRF) and entered into the Electronic Data Capture System by trained clinical research coordinators. To ensure confidentiality, CRFs will be identified with only initials and participation number rather than the participants’ real identities. Besides, all personal information collected from the enrolled participants will be stored by investigators of each hospital in a locked area. An Independent Data Safety Monitoring Board (IDSMB) including one neurologist, one biostatistician and one clinical pharmacologist will be assembled by the lead investigator, with responsibilities including but not limit to safeguard the interests of trial participants, assess the safety and efficacy of the interventions during the trial, monitor the overall conduct of the clinical trial, and provide recommendations about stopping or continuing the trial [26]. An independent biostatistician team selected by the members of IDSMB will perform the interim analysis and a ‘Formal Interim Analysis’ meeting will be held by the IDSMB to review data relating to the treatment efficacy, patient safety and quality of trial conduct. The IDSMB will let the study team know the final decision and the whole team has no access to the interim analysis result. The data managers and study biostatisticians will only access the data for its management and statistical analysis and will not be involved in the clinical management in any regards.

### Sample size estimation

The sample size calculation was based on previous findings, and the proportion of patient with a mRS of 0–2 at 90 days of 41% was estimated for the placebo group, and of 58% was assumed for the glibenclamide-treated group [11]. When half of the planned subjects finish their 90-day follow up an interim analysis will be conducted and the O’Brien - Fleming method will be used to control the type I error. Using nQuery + nTerim 4.0 (Statistical Solutions Ltd., Farmer’s Cross, Cork, Ireland) with a 2-sided χ^2^ test, a two-sided significance level of 0.05, a power of 80%, the O’Brien - Fleming alpha spending function and a dropout rate < 10%, 153 patients are required in each group, for a total sample size of 306.

### Statistical analyses

The primary outcome will be compared between 2 groups using χ^2^ tests, and the confidence interval of the rate difference will be calculated using the Newcombe’s method [27]. Common risk difference for multiway 2 × 2 tables stratified by endovascular treatment and baseline NIHSS score (≥ 14 or < 14) will also be reported. The Mantel-Haenszel estimate, confidence limits, and test for the common risk differences by using Mantel-Haenszel stratum weights and the Sato variance estimator will be used. When half of the planned subjects are recruited and their 90-day follow up are finished, interim analysis will be conducted by a selected biostatistician. O’Brien - Fleming method will be used to control the Type I error. The trial will be stopped early when the primary outcome of the interim analysis significantly favors or contradicts the usage of glibenclamide.

For baseline variables and other outcomes, comparisons between normally distributed continuous variables, expressed as mean ± SD, will be performed using 2-sample t tests; non-normally distributed continuous variables, presented as median and interquartile range, will be analyzed using Wilcoxon rank sum tests. Pearson χ^2^ or Fisher exact tests will be used, as appropriate, for categorical data, which will be expressed as percentages. For secondary outcomes mRS shift and MMP-9, mixed effect model will also be used.

For safety analysis, all adverse events and serious adverse events, including deaths, will be summarized by body systems in terms of frequency, severity, and relatedness to the study drug using the MedDRA codes. Frequencies of adverse events from these two groups will be reported. Pearson χ^2^ or Fisher exact tests will be used, as appropriate, to compare the incidence of adverse events between the two groups.

Subgroups analyses will be performed according to pre-defined design variables, such as: sex, age (≤ 60 years vs. 61–74 years), endovascular treatment, NIHSS grade (4–13 vs. 14–25), time window in rtPA thrombolysis (< 3.5 h vs. 3.5–4.5 h), and study drug treatment time (< 6 h vs. 6–10 h).

The primary and secondary end points will be analyzed based on the modified intention-to-treat (mITT) population which excludes those patients who do not receive treatment or control and has no efficacy outcome data, and those subjects who are diagnosed with posterior circulation occlusion alone after being included. Per-protocol patients who finish the treatment protocol with good compliance, will also be analyzed as a sensitive analysis. Safety analyses will be done in patients receiving allocated treatment. Last observation carried forward method will be used to deal with the missing data of primary outcome, and multiple imputation method will also be used as sensitive analysis. All analyses will be 2-tailed and a significance level of 0.05 will be used for all outcomes. All data analyses will be performed using SAS version 9.4 (SAS Institute, Cary, NC).

### Patient and public involvement

There was no patient or public involvement in the study design.

## Discussion

Intravenous rtPA thrombolysis is still the mainstay of AIS treatment. However, the restricted time window and hemorrhagic transformation related side effects have limited its wide application. Even in patients treated with intravenous rtPA, more than 50% of them continue to have substantial disability [28]. Moreover, thrombolytic therapy substantially increases the risk of hemorrhagic transformation after AIS, probably by disrupting the blood-brain barrier. Researchers have tried to enhance the efficacy of intravenous rtPA thrombolysis by add-on therapy [29, 30], and some benefits have been observed from the selective population [31].

Glibenclamide is a second generation sulfonylurea that has long been used for treating type 2 diabetes mellitus. By directly binding to the sulfonylurea receptor 1 (SUR1) subunits of K-ATP channels, glibenclamide causes the channel closure and results in membrane depolarization in the pancreatic β-cell leading to increased insulin secretion [32]. During the last decade, glibenclamide has repeatedly shown to exert prominent neuroprotection via blocking of the newly synthesized SUR1-transient receptor potential M4 (SUR1-TRPM4) channel [33]. In a rodent model of transient middle cerebral artery occlusion, glibenclamide reduced cerebral edema, infarct volume and mortality by 50% [7]. Findings from other rat models of ischemic stroke and data from independent laboratory further support the significance of glibenclamide in alleviating ischemic injury [8, 34]. Strikingly, glibenclamide attenuated rtPA-related brain injury, with a therapeutic time window of 10 h [13].

A retrospective study in AIS patients with type 2 diabetes mellitus who took sulfonylurea agents both at the time of stroke and during hospitalization had got better outcome than those who did not [10]. Another study reported sulfonylurea agents were associated with less hemorrhagic transformation, indicating that sulfonylurea might function in blood-brain barrier protection [11]. Postmortem examination using specimen from patients with brain infarction supports the notion that sulfonylurea takes effect by blocking the upregulated SUR1 [35].

A double-blind, randomized, placebo-controlled phase 2 trial has been conducted to test the efficacy of intravenous glibenclamide (RP-1127) in preventing brain swelling after large hemispheric infarction (GAMES-RP) [15]. Patients (aged 18–80 years) with a clinical diagnosis of large anterior circulation hemispheric infarction for less than 10 h and baseline diffusion-weighted MRI image lesion volume of 82–300 cm^3^ on MRI were enrolled. Although the study was suspended for funding reason, interim results revealed that glibenclamide was well tolerated and reduced 30-day mortality as well as midline shift [12]. Moreover, glibenclamide decreased the serum concentration of MMP-9, indicating a protective role of glibenclamide in blood-brain barrier. In our preliminary study, we found that a low-dose regimen of oral glibenclamide was well tolerated and was associated with reduced brain edema and consequential severe disability and death [17].

Based on the above data and as a complement to the GAMES-RP study, we conducted this prospective, randomized, double-blind, placebo-controlled, multicenter trial to evaluate the efficacy of glibenclamide in AIS patients treated with intravenous rtPA. Although parenteral glibenclamide had been used in animal studies and the GAMES-RP study (RP-1127), no commercial formulation for clinical use is available in China, so the enteral route was chosen. Glibenclamide is well absorbed from the gastrointestinal tract with its peak plasma concentration occurred 1.5–2 h after ingestion and has a duration of action approximately for 18 h [36]. Besides, oral glibenclamide has been safely used for decades, with limited evidence regarding safety issues, except hypoglycemia [37]. Since its apparent potency may increase at the acidic microenvironment found in ischemic brain [7], a relatively low-dose regimen of glibenclamide would be reasonable and thereby the risk of hypoglycemia will be greatly reduced [15]. Given that poststroke hyperglycemia is common and prolonged in nondiabetic as well as diabetic patients, our low-dose regimen of glibenclamide, which is about 25% of its regular dose for treating diabetes, might not substantially increase the risk of hypoglycemia in the study population. In our preliminary study, only one episode of hypoglycemia was recorded during treatment with the glibenclamide in the nondiabetics (*n* = 15), whereas there was 8 episodes of hypoglycemia in the controlled nondiabetics (*n* = 145) (post-hoc analysis) [17]. The low risk of hypoglycemia in these patients may also be related to our early initiation of nutritional support. Therefore, in the SE-GRACE study, early nutrition support and blood glucose monitoring will be adopted to minimize the risk of hypoglycemia. Because the price of oral glibenclamide is very low, a large number of patients would benefit from this agent if it gets proved to be effective.

In conclusion, this article describes the design and planned analysis used in the SE-GRACE trial for the first publication of the primary outcomes. This approach minimizes the overall risk of bias and data-driven results.

## Supplementary information

**Additional file 1: Supplementary Table.** Study sites and site principal investigators of the SE-GRACE trial.

**Additional file 2.** SPIRIT 2013 Checklist

## Data Availability

Not applicable.

## Abbreviations

AE: Adverse events
AIS: Acute ischemic stroke
BI: Barthel Index
CRF: Case report form
IDSMB: Intendent Data Safety Monitoring Board
IQCODE: Informant Questionnaire on Cognitive Decline in the Elderly
mITT: modified intention-to-treat
MMP-9: Matrix metalloproteinase-9
mRS: modified Rankin Scale
PH: Parenchymal hemorrhagic transformation
rtPA: recombinant tissue plasminogen activator
SUR1: Sulfonylurea receptor 1
SUSAR: Suspected unexpected serious adverse reaction
TRPM4: Transient receptor potential M4

## References

[1] Lozano R, Naghavi M, Foreman K, Lim S, Shibuya K, Aboyans V (2012). Global and regional mortality from 235 causes of death for 20 age groups in 1990 and 2010: a systematic analysis for the global burden of disease study 2010. Lancet..

[2] Thrift AG, Cadilhac DA, Thayabaranathan T, Howard G, Howard VJ, Rothwell PM (2013). Global stroke statistics. Int J Stroke.

[3] Wardlaw JM, Murray V, Berge E, Del ZG (2014). Thrombolysis for acute ischaemic stroke. Cochrane Database Syst Rev.

[4] Alexandrov AV (2010). Current and future recanalization strategies for acute ischemic stroke. J Intern Med.

[5] Rodrigues FB, Neves JB, Caldeira D, Ferro JM, Ferreira JJ, Costa J. Endovascular treatment versus medical care alone for ischaemic stroke: Systematic review and meta-analysis. BMJ. 2016;353:i1754. 10.1136/bmj.i1754 PMC4834754 27091337

[6] Powers WJ, Derdeyn CP, Biller J, Coffey CS, Hoh BL, Jauch EC (2015). 2015 american heart association/American stroke association focused update of the 2013 guidelines for the early management of patients with acute ischemic stroke regarding endovascular treatment. Stroke..

[7] Simard JM, Chen M, Tarasov KV, Bhatta S, Ivanova S, Melnitchenko L (2006). Newly expressed SUR1-regulated NC (Ca-ATP) channel mediates cerebral edema after ischemic stroke. Nat Med.

[8] Simard JM, Yurovsky V, Tsymbalyuk N, Melnichenko L, Ivanova S, Gerzanich V (2009). Protective effect of delayed treatment with low-dose glibenclamide in three models of ischemic stroke. Stroke..

[9] Simard JM, Woo SK, Tsymbalyuk N, Voloshyn O, Yurovsky V, Ivanova S (2012). Glibenclamide-10-h treatment window in a clinically relevant model of stroke. Transl Stroke Res.

[10] Kunte H, Schmidt S, Eliasziw M, Del Zoppo GJ, Simard JM, Masuhr F (2007). Sulfonylureas improve outcome in patients with type 2 diabetes and acute ischemic stroke. Stroke..

[11] Kunte H, Busch MA, Trostdorf K, Vollnberg B, Harms L, Mehta RI (2012). Hemorrhagic transformation of ischemic stroke in diabetics on sulfonylureas. Ann Neurol.

[12] Sheth KN, Elm JJ, Molyneaux BJ, Hinson H, Beslow LA, Sze GK (2016). Safety and efficacy of intravenous glyburide on brain swelling after large hemispheric infarction (GAMES-RP): a randomised, double-blind, placebo-controlled phase 2 trial. Lancet Neurol.

[13] Simard JM, Geng Z, Silver FL, Sheth KN, Kimberly WT, Stern BJ (2012). Does inhibiting Sur1 complement rt-PA in cerebral ischemia?. Ann N Y Acad Sci.

[14] Powers WJ, Rabinstein AA, Ackerson T, Adeoye OM, Bambakidis NC, Becker K (2018). 2018 guidelines for the early management of patients with acute ischemic stroke: a guideline for healthcare professionals from the american heart association/American stroke association. Stroke..

[15] Sheth KN, Elm JJ, Beslow LA, Sze GK, Kimberly WT. Glyburide advantage in malignant edema and stroke (GAMES-RP) trial: rationale and design. Neurocrit Care. 2016;24:132–9. 10.1007/s12028-015-0189-7 26268138

[16] Sheth KN, Kimberly WT, Elm JJ, Kent TA, Mandava P, Yoo AJ (2014). Pilot study of intravenous glyburide in patients with a large ischemic stroke. Stroke..

[17] Huang K, Hu Y, Wu Y, Ji Z, Wang S, Lin Z (2019). Exploratory analysis of oral glibenclamide in acute ischemic stroke. Acta Neurol Scand.

[18] Chinese Society of Neurology, Chinese Stroke Society (2018). Chinese guidelines for diagnosis and treatment of acute ischemic stroke 2018. Chin J Neurol.

[19] McClave SA, Taylor BE, Martindale RG, Warren MM, Johnson DR, Braunschweig C (2015). Guidelines for the provision and assessment of nutrition support therapy in the adult critically ill patient. Jpen-Parenter Enter.

[20] Vahedi K, Hofmeijer J, Juettler E, Vicaut E, George B, Algra A (2007). Early decompressive surgery in malignant infarction of the middle cerebral artery: a pooled analysis of three randomised controlled trials. Lancet Neurol.

[21] Harrison JK, Fearon P, Noel-Storr AH, McShane R, Stott DJ, Quinn TJ. Informant questionnaire on cognitive decline in the elderly (IQCODE) for the diagnosis of dementia within a secondary care setting. Cochrane Db Syst Rev. 2015. 10.1002/14651858.CD010772.pub2 25754745

[22] McGovern A, Pendlebury ST, Mishra NK, Fan Y, Quinn TJ (2016). Test accuracy of informant-based cognitive screening tests for diagnosis of dementia and multidomain cognitive impairment in stroke. Stroke..

[23] Duffy L, Gajree S, Langhorne P, Stott DJ, Quinn TJ (2013). Reliability (inter-rater agreement) of the Barthel index for assessment of stroke survivors: systematic review and meta-analysis. Stroke..

[24] Berger C, Fiorelli M, Steiner T, Schabitz WR, Bozzao L, Bluhmki E (2001). Hemorrhagic transformation of ischemic brain tissue: asymptomatic or symptomatic?. Stroke..

[25] von Kummer R (2002). Brain hemorrhage after thrombolysis: good or bad?. Stroke..

[26] Wiberg S, Hassager C, Thomsen JH, Frydland M, Høfsten DE, Engstrøm T (2016). GLP-1 analogues for neuroprotection after out-of-hospital cardiac arrest: study protocol for a randomized controlled trial. Trials..

[27] Newcombe RG (1998). Interval estimation for the difference between independent proportions: comparison of eleven methods. Stat Med.

[28] Dharmasaroja P, Muengtaweepongsa S (2016). Outcomes of patients with large middle cerebral artery infarct treated with and without intravenous thrombolysis. J Neurosci Rural Pract.

[29] Chamorro A, Amaro S, Castellanos M, Segura T, Arenillas J, Marti-Fabregas J (2014). Safety and efficacy of uric acid in patients with acute stroke (URICO-ICTUS): a randomised, double-blind phase 2b/3 trial. Lancet Neurol.

[30] Adeoye O, Sucharew H, Khoury J, Tomsick T, Khatri P, Palesch Y (2015). Recombinant tissue-type plasminogen activator plus eptifibatide versus recombinant tissue-type plasminogen activator alone in acute ischemic stroke. Stroke..

[31] Llull L, Laredo C, Renú A, Pérez B, Vila E, Obach V (2015). Uric acid therapy improves clinical outcome in women with acute ischemic stroke. Stroke..

[32] Malek R, Davis SN (2016). Pharmacokinetics, efficacy and safety of glyburide for treatment of gestational diabetes mellitus. Expert Opin Drug Metab Toxicol.

[33] Simard JM, Sheth KN, Kimberly WT, Stern BJ, Del ZG, Jacobson S (2014). Glibenclamide in cerebral ischemia and stroke. Neurocrit Care.

[34] Wali B, Ishrat T, Atif F, Hua F, Stein DG, Sayeed I (2012). Glibenclamide administration attenuates infarct volume, hemispheric swelling, and functional impairments following permanent focal cerebral ischemia in rats. Stroke Res Treat.

[35] Mehta RI, Ivanova S, Tosun C, Castellani RJ, Gerzanich V, Simard JM (2013). Sulfonylurea receptor 1 expression in human cerebral infarcts. J Neuropathol Exp Neurol.

[36] Feldman JM (1985). Glyburide: a second-generation sulfonylurea hypoglycemic agent. History, chemistry, metabolism, pharmacokinetics, clinical use and adverse effects. Pharmacotherapy..

[37] Song R, Chen L, Chen Y, Si X, Liu Y, Liu Y (2017). Comparison of glyburide and insulin in the management of gestational diabetes: a meta-analysis. PLoS One.

