# Supplementary material for: Safety and efficacy of glibenclamide combined with rtPA in acute cerebral ischemia with occlusion/stenosis of anterior circulation (SE-GRACE): study protocol for a randomized controlled trial
Source: BMC Neurol. 2020 Jun 11;20:239. doi: 10.1186/s12883-020-01823-z (PMC7291425; doi:10.1186/s12883-020-01823-z)
Supplement: Supplementary file 1 — Additional file 1: Supplementary Table. Study sites and site principal investigators of the SE-GRACE trial. [file 12883_2020_1823_MOESM1_ESM.docx]

**Supplementary Table**. Study sites, site principal investigators, and local ethics committee that provided ethics approval of the SE-GRACE trial.

| **Study sites** | **Site principal investigators** | **Full name of the local ethics committee** |
| --- | --- | --- |
| Nanfang Hospital, Southern Medical University | Zhong Ji | Medical Ethics Committee of Nanfang Hospital |
| Heyuan People’s Hospital | Yunqiang Huang | Medical Ethics Committee of Heyuan People’s Hospital |
| Huadu district People's Hospital | Guangning Li | Medical Ethics Committee of Huadu district People's Hospital |
| The First Affiliated Hospital of Wenzhou Medical University | Xu Zhang | Medical Ethics Committee of the First Affiliated Hospital of Wenzhou Medical University |
| Maoming People’s Hospital | Zhi Yang | Medical Ethics Committee of Maoming People’s Hospital |
| Maoming Hospital of Traditional Chinese Medicine | Wenguo Huang | Medical Ethics Committee of Maoming Hospital of Traditional Chinese Medicine |
| Haikou People’s Hospital | Guoshuai Yang | Medical Ethics Committee of Haikou People’s Hospital |
| Hainan Hospital of Traditional Chinese Medicine | Guohu Weng | Medical Ethics Committee of Hainan Hospital of Traditional Chinese Medicine |
